# Dermatophilosis among men who have sex with men, Stockholm, Sweden, March to June, 2026

**DOI:** 10.2807/1560-7917.ES.2026.31.25.2600520

**Published:** 2026-06-25

**Authors:** Finn Filén, Elin Loo, Karin Haij Bhattarai

**Affiliations:** 1 Department of Infectious Diseases/Venhälsan, Södersjukhuset, Stockholm, Sweden; 2 Department of Clinical Science and Education, Södersjukhuset, Karolinska Institutet, Stockholm, Sweden; 3 Department of Clinical Microbiology, Karolinska University Hospital, Stockholm, Sweden

**Keywords:** Dermatophilus congolensis, dermatophilosis, MSM, sexually transmitted infection, PrEP

## Abstract

*Dermatophilus congolensis* is traditionally regarded as a zoonotic skin pathogen. Recent European reports suggest possible transmission among men who have sex with men (MSM). We describe four microbiologically confirmed cases in Stockholm, Sweden, in 2026. All occurred in MSM using HIV pre-exposure prophylaxis and without contacts with animals. Infections were probably acquired in Sweden, Japan and Spain. These findings support transmission within sexual networks and suggest that dermatophilosis may be under-recognised in sexual health settings.

Transmission of *Dermatophilus congolensis* among men who have sex with men (MSM) has recently been described in several European countries [8]. We report four cases diagnosed in Stockholm, Sweden, during the period March to June 2026.

## Case descriptions

All four patients were men who have sex with men (MSM) using emtricitabine/tenofovir disoproxil as HIV pre-exposure prophylaxis (PrEP). During case investigations, each patient reported potential exposures before symptom onset, including attendance at sex clubs, gay saunas or spas where environmental exposure, sexual contact or both may have occurred. None of the patients reported contact to livestock or domestic animals. None had chronic skin disease or immunosuppression, and none developed systemic symptoms. Secondary cases were not identified. We present exposure histories and symptom timelines for each case.

### Case 1

A man in his 60s presented with rapidly progressive papular and pustular skin lesions involving the anterior and lateral aspects of the lower abdomen the day after visiting a sex club in Sweden in March 2026, where he had used a hot tub. The initial clinical suspicion was pseudomonal folliculitis, and empirical treatment with ciprofloxacin 500 mg twice daily was initiated. Microbiological culture subsequently identified *Dermatophilus congolensis*. Because clinical response to ciprofloxacin was insufficient, treatment was changed to doxycycline 100 mg twice daily for 7 days, resulting in marked improvement within a few days and complete resolution without recurrence.

### Case 2

**Figure 1:**
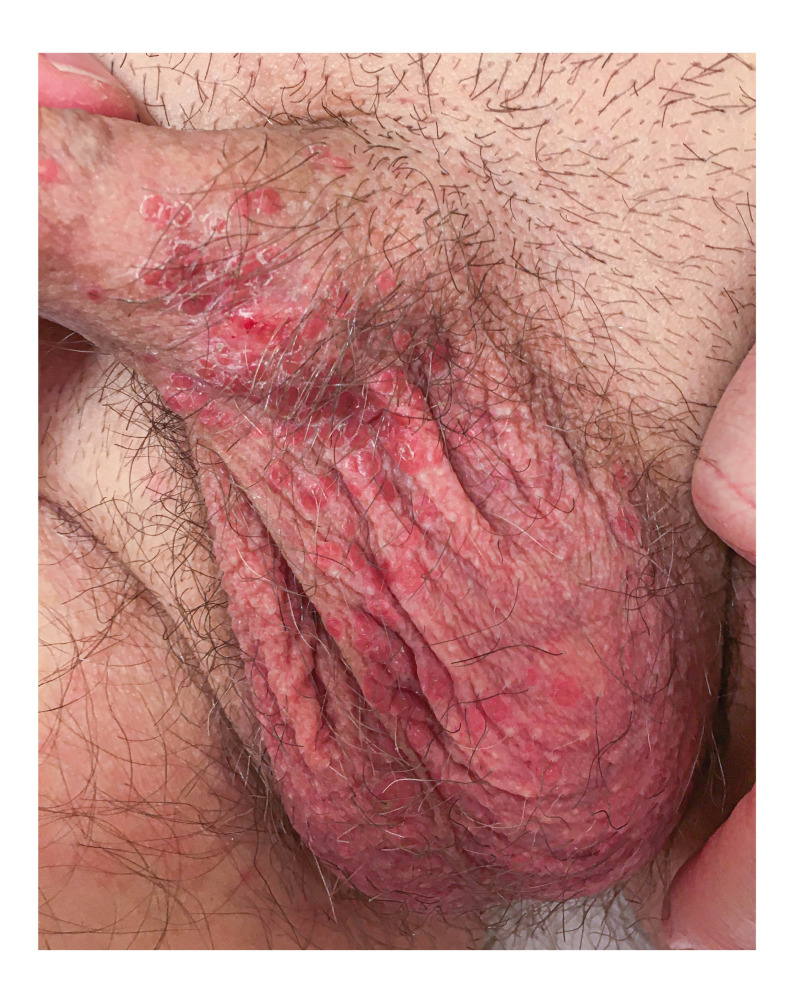
Dermatophilosis scrotum, Case 2, Stockholm, 2026

A man in his 50s developed a papular eruption on the scrotum while travelling in Japan in April 2026, during a 3-week visit (Figure 1). Lesions first appeared 2 days before returning to Sweden.

Papular lesions on the scrotum progressing to scaling, with focal annular scaling at the periphery of individual papules. Photograph obtained 3 weeks after symptom onset.

The lesions gradually spread to the penile shaft, perineum and intergluteal cleft, becoming increasingly scaly over time. He reported multiple recent sexual contacts in Japan, as well as visits to public baths and spa facilities over several weeks. He did not report any further sexual or environmental exposures after lesion onset, neither during the remainder of his stay nor after his return to Sweden. Initial treatment with ciprofloxacin 500 mg twice daily for 7 days was prescribed because pseudomonal folliculitis was suspected. Near completion of therapy, preliminary microbiological results demonstrated *D. congolensis*. As the lesions had not completely resolved, doxycycline 100 mg twice daily for 7 days was added, with clinical improvement within a few days and complete recovery. No recurrence occurred.

### Case 3

**Figure 2:**
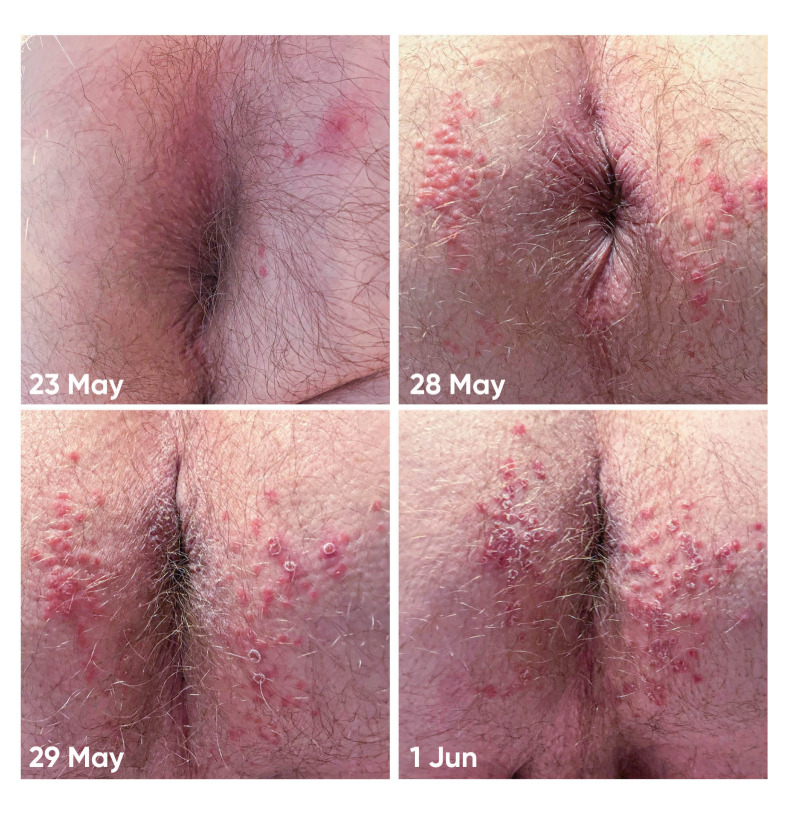
Dermatophilosis progression, Case 3, Stockholm, 2026

A man in his 40s developed papular and subsequently scaly lesions in the intergluteal cleft following attendance at gay saunas in Barcelona over several weeks in 2026 (Figure 2). Based on increasing awareness of recently reported European cases, *D. congolensis* infection was suspected early during the case investigation. Oral amoxicillin 500 mg three times daily for 1 week was prescribed. The patient reported substantial improvement within 24 h and complete clinical resolution within 48 h, although the treatment course was completed as planned. No recurrence was observed. The patient documented the progression of lesions.

Perianal lesions initially presenting as papules and subsequently developing annular scaling at their margins of individual papules. Serial photographs over 8 days; the patient was clinically assessed the following day.

### Case 4

A man in his 40s presented with several days of a perianal rash without associated pain or pruritus. Additional lesions had recently appeared in the groin. The symptoms had started approximately 1 week after both a new sexual contact and attendance at a sex club in Sweden which included a pool. He did not report any systemic symptoms.

On examination, multiple 2–3 mm perianal papules and pustules were observed, with mild surrounding hyperkeratosis, as well as two erythematous papules on the inner thigh. Direct microscopy of lesion material performed at the clinic, using methylene blue staining, demonstrated filamentous structures compatible with *D. congolensis*. Culture showed Gram-positive rods highly suggestive of *D. congolensis*. Topical fusidic acid was prescribed. At follow-up 4 days later, the lesions showed partial clinical improvement but had not completely resolved. No further follow-up data were available.

## Microbiological findings^†^

**Figure 3:**
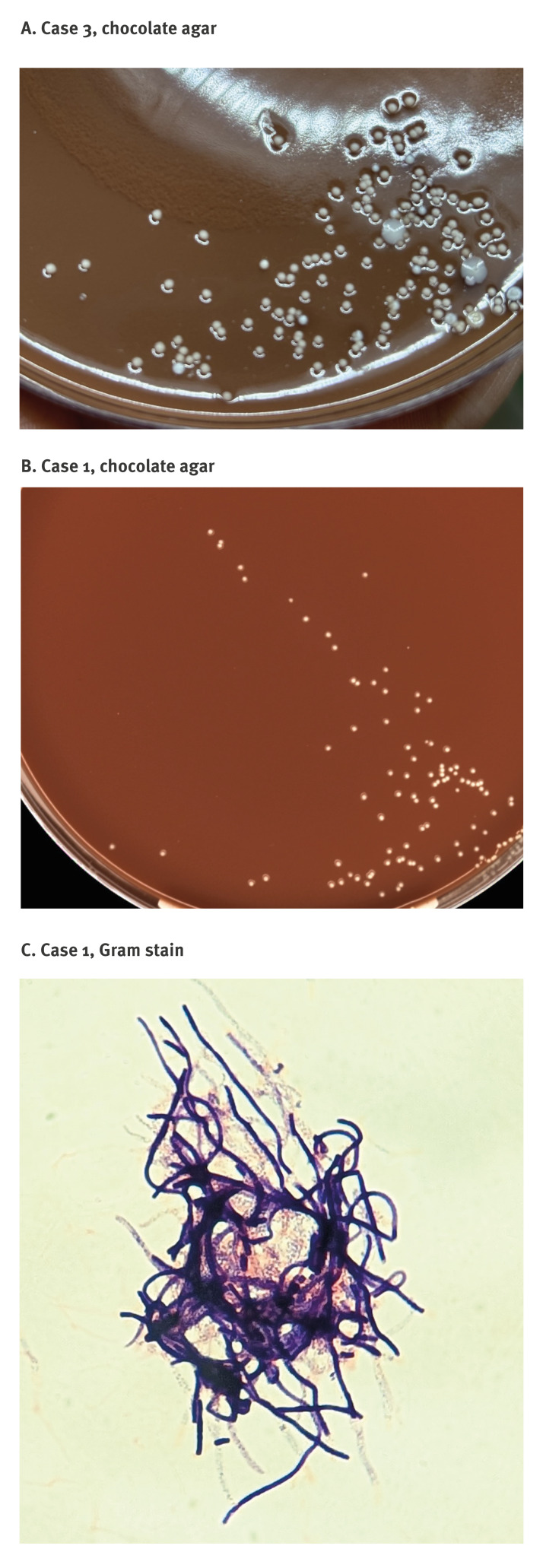
Culture and microscopic findings of *Dermatophilus congolensis* from clinical cases, Stockholm, Sweden, 2026

For each patient, we cultured swabs from the lesions on non-selective media, including horse blood agar incubated under aerobic conditions, chocolate agar at 5% CO_2_ and on FAA or blood agar incubated under anaerobic conditions, using the Kiestra work cell automation system (Becton Dickinson AB, Sweden). After 36 h, growth was most prominent on chocolate agar, presenting as grey-yellow, dry, adherent colonies with a pearl-like appearance (Figure 3). Colonies were hardly visible after incubated under anaerobic conditions. In two cases, concomitant commensal skin flora complicated recognition on digital image analysis, whereas manual inspection highlighted the characteristic adherent, movable, pearl-like colonies. Gram staining revealed long filamentous Gram-positive bacilli in clusters (Figure 3). A MALDI-TOF mass spectrometry (Biotyper Sirius one IVD, Bruker Daltonik, Germany) did not yield reliable identification despite available spectra in the database. Species-level confirmation was achieved in two cases by sequencing 16S rDNA variable regions V1V2 and V3V4 (Ion Torrent, Thermo Fisher Scientific, United States). The sequences are appended in the Supplement. Isolates showed a sequence identity of 99.7–100% against *D. congolensis* reference sequences in the Genome Taxonomy Database. Although 16S rDNA sequencing confirmed the species identity, the generated sequences do not provide sufficient resolution for phylogenetic comparison with previously reported European isolates. Whole genome sequencing will therefore be required to assess genetic relatedness and investigate possible epidemiological links. No additional wound pathogens were identified.

Panel A: Case 3, chocolate agar showing grey-yellow, dry, adherent colonies with a pearl-like appearance. Panel B: Case 1, chocolate agar showing grey-yellow, dry, adherent colonies with a pearl-like appearance. Panel C: Case 1, Gram stain showing long filamentous Gram-positive bacilli in clusters^†^.

## Discussion

*Dermatophilus congolensis* is a globally distributed Gram-positive actinomycete and veterinary pathogen. Human infection has classically been described as a zoonosis causing pruritic pustular or crusted skin lesions after animal exposure [1-4]. Sporadic cases have also been reported in returning travellers, suggesting that exposure may occur outside traditional livestock-associated settings [5]. Recent reports from Spain and France describe suspected sexual transmission of *D. congolensis* infection among MSM without animal exposure [6,7]. A recent rapid risk assessment from the European Centre for Disease Prevention and Control identified clusters across several European countries and concluded that human-to-human transmission through close physical contact is the most likely route of spread [8]. The four Swedish cases presented here share several notable similarities with recently published European cases. As reported in France and Spain, none of our patients reported livestock or animal exposure, and none developed systemic symptoms. Clinical manifestations were initially interpreted as pseudomonal folliculitis in two patients because they had used hot tubs before symptom onset, contributing to underdiagnosis. The clinical images included in this report illustrate some of the presentations observed in our patients and may help familiarise clinicians with this emerging presentation. In early stages, the lesions may also mimic early herpes simplex or mpox, particularly in the genital or perianal region, leading to diagnostic uncertainty. Figure 2, particularly the second image (28 May), illustrates the diagnostic difficulties; scaling is not yet present, and the sudden appearance of papules could be misinterpreted as a viral infection.

The anatomical distribution of lesions, involving genital, perineal, intergluteal and truncal areas, together with the temporal relationship to sexual exposures, is compatible with transmission through intimate skin-to-skin contact. However, transmission through communal wet environments cannot be excluded. In this case series, the small number of patients and uncertainty regarding the timing and number of potential exposures preclude any conclusions regarding the incubation period of human dermatophilosis.

Although this limited case series does not permit conclusions regarding optimal antimicrobial therapy, the treatment responses were broadly consistent with the recently reported Spanish and French clusters, where short courses of β-lactams and doxycycline appeared clinically effective in most patients. In our series, particularly rapid clinical resolution was observed following amoxicillin treatment in one patient. Acquired tetracycline resistance, including identification of the tet(Z) resistance gene, has been reported in veterinary *D. congolensis* isolates, although its relevance for treatment of human infection remains unclear [9,10].

## Conclusion

Human dermatophilosis may be an emerging infection within certain MSM sexual networks. Clinicians working in sexual health services should consider *D. congolensis* infection in MSM presenting with papular, pustular or scaly lesions, particularly among patients who have visited sex clubs, saunas or similar venues. Further microbiological and epidemiological investigations are needed to better understand reservoirs, transmission routes and the possible emergence of dermatophilosis within European sexual networks. One case occurred in temporal association with recent travel to Japan, suggesting that similar transmission networks may extend beyond Europe.

## Data Availability

No datasets were generated beyond the clinical and microbiological data described in this report. Sequences generated in relation to this work are appended in the Supplement.

## ^†^Author's correction

At the request of the authors, several corrections were made on 1 July 2026. In the chapter Microbiological findings, the original text  

*For each patient, we cultured swabs from the lesions on non-selective media, including blood agar incubated under aerobic and anaerobic conditions and chocolate agar at 5% CO2 using the Kiestra work cell automation system (Becton Dickinson AB, Sweden). After 48 h, growth was most prominent on chocolate agar, presenting as grey-yellow, dry, adherent colonies with a pearl-like appearance (Figure 3). On blood agar, colonies were grey-yellow. In two cases, concomitant commensal skin flora complicated recognition on digital image analysis, whereas manual inspection highlighted the characteristic adherent, movable, pearl-like colonies. Gram staining revealed filamentous, branching Gram-positive bacilli with transverse and longitudinal septations*

was changed to read

*For each patient, we cultured swabs from the lesions on non-selective media, including* ***horse*** *blood agar incubated under* ***aerobic*** *conditions, chocolate agar at 5% CO2 and* ***on FAA or blood agar incubated under anaerobic conditions*** *, using the Kiestra work cell automation system (Becton Dickinson AB, Sweden). After* ***36 h*** *, growth was most prominent on chocolate agar, presenting as grey-yellow, dry, adherent colonies with a pearl-like appearance (Figure 3).* ***Colonies were hardly visible after incubated under anaerobic conditions*** *. In two cases, concomitant commensal skin flora complicated recognition on digital image analysis, whereas manual inspection highlighted the characteristic adherent, movable, pearl-like colonies. Gram staining revealed* ***long filamentous Gram-positive bacilli in clusters*** *.*

The footnote of Figure 3 was changed accordingly from* filamentous, branching Gram-positive bacilli with transverse and longitudinal septations *to* long filamentous Gram-positive bacilli in clusters.*

## Supplementary Data

63000 Supplement
