# Author’s correction for Euro Surveill. 2026;31(25)

**DOI:** 10.2807/1560-7917.ES.2026.31.26.260702c

**Published:** 2026-07-02

**Authors:** Eurosurveillance editorial team

**Affiliations:** 1 European Centre for Disease Prevention and Control (ECDC), Stockholm, Sweden

At the request of the authors, several corrections were made in the article ‘Dermatophilosis among men who have sex with men, Stockholm, Sweden, March to June, 2026’ by Filen et al., originally published on 25 June 2026: 

In the chapter Microbiological findings, the original text 

*For each patient, we cultured swabs from the lesions on non-selective media, including blood agar incubated under aerobic and anaerobic conditions and chocolate agar at 5% CO2 using the Kiestra work cell automation system (Becton Dickinson AB, Sweden). After 48 h, growth was most prominent on chocolate agar, presenting as grey-yellow, dry, adherent colonies with a pearl-like appearance (Figure 3). On blood agar, colonies were grey-yellow. In two cases, concomitant commensal skin flora complicated recognition on digital image analysis, whereas manual inspection highlighted the characteristic adherent, movable, pearl-like colonies. Gram staining revealed filamentous, branching Gram-positive bacilli with transverse and longitudinal septations*

was changed to read 

*For each patient, we cultured swabs from the lesions on non-selective media, including horse blood agar incubated under aerobic conditions, chocolate agar at 5% CO2 and on FAA or blood agar incubated under anaerobic conditions, using the Kiestra work cell automation system (Becton Dickinson AB, Sweden). After 36 h, growth was most prominent on chocolate agar, presenting as grey-yellow, dry, adherent colonies with a pearl-like appearance (Figure 3). Colonies were hardly visible after incubated under anaerobic conditions. In two cases, concomitant commensal skin flora complicated recognition on digital image analysis, whereas manual inspection highlighted the characteristic adherent, movable, pearl-like colonies. Gram staining revealed long filamentous Gram-positive bacilli in clusters.*

The footnote of Figure 3 was changed accordingly from *filamentous, branching Gram-positive bacilli with transverse and longitudinal septations* to long *filamentous Gram-positive bacilli in clusters*.

These changes were made on 1 July 2026. 

