# Supplementary material for: Dermatophilosis among men who have sex with men, Stockholm, Sweden, March to June, 2026
Source: Euro Surveill. 2026 Jun 25;31(25):2600520. doi: 10.2807/1560-7917.ES.2026.31.25.2600520 (PMC13309758; doi:10.2807/1560-7917.ES.2026.31.25.2600520)
Supplement: Supplement [file 26-00520_FILEN_Supplement.pdf]

Supplement S1. Representative 16S rDNA V1V2 and V3V4 sequences from *Dermatophilus congolensis* isolates

This supplementary material is hosted by *Eurosurveillance* as supporting information alongside the article "Dermatophilosis among men who have sex with men, Stockholm, Sweden, March to June, 2026", on behalf of the authors, who remain responsible for the accuracy and appropriateness of the content. The same standards for ethics, copyright, attributions and permissions as for the article apply. Supplements are not edited by *Eurosurveillance* and the journal is not responsible for the maintenance of any links or email addresses provided therein.

Representative genome sequences of variable regions of 16S rDNA sequenced in Case 1:

V1V2:
TCTGGGCCGTGTCTCAGTCCCAGTGTGGCCGTTCACCCTCTCAGGCCGGCTACCCGTCGTCGCCTTGGTGAGCCG
TTACCTCACCAACAAGCTGATAGGCCGCGAGCACATCCCCCACCGAAAAAACTTTCCACCAAACCTCATGCGAGGA
AAGGTCATATTCAGTATTAGACCCCGTTTCCGGGGCTTATCCCAAAGTGAGGGGCAGATTACTCACGTGTTACTCA
CCCGTTCGCCACTAATCCACCCAGCAAGCTGGGCTTCATCGTTCGACTTGCATGTGTTAAGCACGCCGCCAGCGTT
CGTC
V3V4:
TGTTCGCTACCCATGCTTTCGCTTCTCAGCGTCAGTAATGGCCCAGAGACCTGCCTTCGCCATCGGTGTTCCTCCT
GATATCTGCGCATTTCACCGCTACACCAGGAATTCCAGTCTCCCCTACCACACTCTAGCCTGCCCGTACCCACTGCA
CGTCCAGGGTTAAGCCCTGGATTTTCACAGCAGACGCGACAAACCGCCTACAAGCTCTTTACGCCCAATAATTCCG
GACAACGCTCGCACCCTACGTATTACCGCGGCTGCTGGCACGTAGTTAGCCGGTGCTTCTTCTGCAGGTACCGTCA
CTTTCGCTTCTCCCCTGCTGAAAGAGGTTTACAACCCGAAGGCCTTCATCCCTCACGCGGCGTCGCTGCATCAGGC
TTTCGCCCATTGTGCAATATTCCCCAC
